# Associations of Prenatal and Perinatal Factors with Cortisol Diurnal Pattern and Reactivity to Stress at Preschool Age Among Children Living in Poverty

**DOI:** 10.4274/jcrpe.1685

**Published:** 2015-06-03

**Authors:** Maha E. Elhassan, Alison L. Miller, Delia M. Vazquez, Julie C. Lumeng

**Affiliations:** 1 Michigan University Faculty of Medicine, Department of Pediatrics, Division of Endocrinology, Michigan, USA; 2 Michigan University Faculty of Medicine, School of Public Health, Department of Health Behavior and Health Education, Michigan, USA; 3 Michigan University Center for Human Growth and Development, Michigan, USA; 4 Michigan University Faculty of Medicine, Department of Psychiatry, Michigan, USA; 5 Michigan University Faculty of Medicine, Department of Pediatrics, Division of Child Behavioral Health, Michigan, USA; 6 Michigan University, School of Public Health, Department of Environmental Health Sciences, Michigan, USA

**Keywords:** HPA axis, pre-pregnancy BMI, pregnancy weight gain, preschool age

## Abstract

**Objective:**

To examine the association of pre- and perinatal factors with diurnal cortisol pattern and reactivity to a stressor at preschool age among children living in poverty.

**Methods:**

Preschool aged children (n=275) provided saliva samples 3 times per day for 3 days to assess circadian rhythmicity (intercept and slope reflected diurnal pattern) and during a behavioral stress elicitation protocol to measure reactivity (5 samples before, during and after the stressor). Pre- and perinatal predictors were pregnancy weight gain, pre-pregnancy body mass index (BMI), infant birth weight z-score and gestational age. We ran 7 linear regression models predicting each of the cortisol outcomes including all pre- and perinatal predictors and covariates simultaneously.

**Results:**

Greater pregnancy weight gain predicted higher morning cortisol [b=0.020 (SE 0.007), p=0.003]. Greater pregnancy weight gain also predicted higher cortisol at recovery from the stressor in girls only [β=0.002 (SE 0.001), p=0.036]. There was no association of pre-pregnancy BMI with any cortisol outcome. Higher birth weight z-score predicted higher morning cortisol in the total sample [β=0.134 (SE 0.066, p=0.043]. Greater gestational age predicted lower cortisol during peak stress in the sample who underwent cortisol reactivity testing [β=-0.015 (SE 0.007), p=0.032] and in boys [β=-0.032 (SE 0.014), p=0.027].

**Conclusion:**

Pre- and perinatal factors are associated with cortisol patterning in offspring at preschool age. The implications for child health require additional studies.

## INTRODUCTION

Understanding pre- and perinatal factors that shape cortisol diurnal pattern and cortisol reactivity to a stressor is important because cortisol patterning is associated with a wide variety of physical and mental health outcomes (1). Few studies have examined potential pre- and perinatal predictors of cortisol patterning in children and their results have been inconsistent. The single study that examined maternal pregnancy weight gain found no association with the child’s cortisol level in a fasting plasma sample at age 8.5 years (2). No studies have examined maternal pre-pregnancy body mass index (BMI) as a potential predictor of cortisol patterning in the child. Of the studies examining the association of infant birth weight with cortisol patterning, some found an inverse association with hypothalamic-pituitary-adrenal (HPA) axis activity (3,4,5,6,7), others found a positive association (8,9), while others showed no association (10,11,12,13). The single study examining the association of infant gestational age with HPA axis functioning showed no association with saliva cortisol in adults (6). Interpretation of findings from these studies is limited by the fact that HPA axis outcomes were tested at widely varying ages, including late adulthood (5,6,7,8,10,12), childhood (3,4,11,13) and infancy (9). Interpretation is also limited due to varying cortisol measurement approaches, including serum (4,5,9,10,12,13), saliva (6,8,11) and urinary metabolites (3,7). In addition, most prior studies have been in European cohorts and there was broad variability in how cortisol patterning was characterized.

Thus, to our knowledge, no study has examined the independent associations of maternal pregnancy weight gain and pre-pregnancy BMI, infant’s birth weight and infant’s gestational age, with cortisol diurnal rhythm or cortisol reactivity to a stressor. In the present study, we tested the association of pre- and perinatal factors with salivary cortisol diurnal pattern and cortisol reactivity to a stressor in a racially and ethnically diverse sample of low-income United States preschoolers.

## METHODS

Children attending Head Start, a free, federally-funded preschool program for low-income children and their primary caregiver and legal guardian were invited to participate in a study about children’s cortisol diurnal pattern and reactivity to a stressor. It was planned that the children participate in the protocol examining reactivity to a stressor on average 6 months after they had participated in the protocol examining cortisol diurnal pattern. Average age of participants in the diurnal cortisol sample was 51 months and in the reactivity sample was 57 months. Exclusion criteria were; parent with ≥4-year college degree; parent or child not English-speaking; child in foster care, with food allergies, significant medical problems or perinatal complications, gestational age <35 weeks or use of medication known to affect cortisol. The study was approved by the University of Michigan Institutional Review Board. Written informed consent was provided by parents and age-appropriate assent was obtained from children; families were compensated for their time. The sample described in this report includes participants with complete data for the predictor, outcome and all covariates in this analysis (n=275 for the diurnal cortisol data and n=179 for the cortisol reactivity data).

Primary caregivers reported children’s sex, birth date, race/ethnicity (categorized for this report as non-Hispanic white vs. not), family structure (single parent vs. not) and primary caregiver education (high school diploma or less vs. more than high school). Children were weighed and measured and BMI calculated concurrent with each saliva collection protocol (diurnal and reactivity).

Primary predictors in our models included infant birth weight, infant gestational age, maternal pre-pregnancy BMI and weight gain during the pregnancy. In order to evaluate pre-pregnancy BMI, mothers were asked, “Just before you got pregnant with (child’s name), how much did you weigh?” Answers were recorded in pounds and converted to kilograms. Mothers’ heights were measured during the study protocol. BMI was calculated. Mothers were also asked, “How much weight did you gain during this pregnancy?”. Answers were recorded in pounds and converted to kilograms. Mothers were asked to report their due date and the child’s actual birth date. From these dates, gestational age was calculated. Mothers were also asked to report their infant’s birth weight, which they provided in pounds and ounces; these were converted to kilograms.

It is known that salivary cortisol is highly correlated with free serum cortisol (14). To capture the pattern of diurnal cortisol secretion, children provided three saliva samples per day for three consecutive days. These were collected on arrival to preschool but before the child ate breakfast at about 8:30 AM; before lunch, at about 11:30 AM; and then at the child’s home, at about 4:30 PM. To capture cortisol reactivity to a stressor, five saliva samples were obtained across a two-hour period before, during and after a stress elicitation procedure.

The stress elicitation procedure was as follows. The child was brought to a room separate from the parent and was first engaged in calming by free play with the examiner for 20 minutes. The child then participated in four challenge tasks. Each task was designed to elicit a mild to moderate level of stress, with tasks including a negative social evaluation component, which is a particularly robust elicitor of cortisol reactivity. The child first rated six prizes from most preferred (e.g., toy car or doll) to least preferred (e.g., broken comb or deflated ball) and was told he or she could have the most preferred prize as a gift later. The examiner then removed the prize from the room.

During the first challenge task, Perfect Circles, the examiner instructed the child to draw a “perfect circle”. For 3.5 minutes, the examiner critiqued each circle the child drew, explaining that the circle was not quite perfect enough and telling the child to “keep trying.” The examiner ultimately told the child that the final circle was “pretty good” and moved on to the next task. During the second challenge task, Puzzles, children were instructed to persist at solving a wooden puzzle that, though age-appropriate, contained two incorrect pieces, making it impossible to solve. After persisting for 3 minutes, the child was told, “we’re out of time on that one” and the puzzle was removed. Then, children were instructed to solve a puzzle that was designed for older children and therefore too difficult because it was age-inappropriate. After 4 minutes, the child was again told that time was up. No child was able to solve the puzzle. The examiner did not provide help, encouragement, or reassurance, but at the conclusion of the Puzzles task acknowledged that the puzzles were “hard”. The examiner then told the child that he or she could now have the previously selected prize, but that the examiner first needed to gift wrap it. During this third challenge task, Gift Wrap/Wait, the child waited for 1.5 minutes while the examiner pretended to wrap the gift behind a screen by crinkling paper. For the fourth challenge task, Disappointing Gift, the examiner presented the child with the box containing the gift. However, instead of containing the child’s preselected, most preferred prize (e.g. toy car or doll), the box instead contained the least preferred prize (e.g. broken comb or deflated ball). The child opened the gift box and the examiner remained unresponsive for 30 seconds while the child reacted to the disappointing gift. After 30 seconds, the examiner “realized” the mistake, apologized for the “mistake” and retrieved the “correct” preferred prize for the child, which the child was allowed to take home as a gift. For 40 more minutes, the child was given the choice of engaging in quiet free play with the examiner or watching an age-appropriate children’s movie.

Saliva was sampled at five points during the protocol: (1) 20 minutes after room entry, to reflect cortisol prior to beginning the study session; (2) 30 minutes after room entry, (10 minutes into the free play period) prior to beginning the challenge tasks; (3) at 10 minutes after receipt of the gift; (4) at 20 minutes after receipt of the gift and (5) at 40 minutes after receipt of the gift. Data points reflect baseline cortisol prior to the behavioral stressor, cortisol increase in response to the stressor and cortisol decline after the stressor. For both the diurnal and reactivity protocols, saliva diaries were collected and carefully recorded anything unusual that happened to the child that day; if the child was ill; whether the child had an unusual sleep pattern; what time the child woke up that day; what time the child ate prior to sample collection and whether the child had taken any medication the day of collection (prescribed or over the counter).

Saliva was stored at -20 ˚C until extracted and assayed in duplicate using an Expanded Range High Sensitivity Salivary Cortisol Enzyme Immunoassay Kit (Salimetrics LLC, PA, USA). For the diurnal samples, the detection limit was 0.007 μg/dL and intra and inter-assay coefficients of variation of 7%, respectively. For the reactivity samples, the detection limit was 0.003 μg/dL and the intra and inter-assay coefficients of variation were 4.6% and 5.5%, respectively. Salivary cortisol concentrations vary by age, gender and time of the day. The mean 8 am morning salivary cortisol reported in children is 0.301 µg/dL with a low value of 0.152 µg/dL [-1 standard deviation (SD)] and a high of 1.176 µg/dL (+2 SD) (15).

Data analyses were performed using STATA 13.0. Univariate and bivariate statistics were used to describe the sample. The cortisol diurnal pattern was captured as an intercept and slope. Specifically, a linear representation of the diurnal cortisol pattern was generated using the log transformed cortisol as the outcome and the time (since awakening) at which the cortisol sampling occurred as the independent variable. From the linear trajectory, an intercept and a slope could be captured (methods were reported in detail elsewhere (16). The cortisol intercept represents the morning peak and the slope represents the rate at which the cortisol declined over the course of the day. Cortisol reactivity was evaluated as an outcome by examining each of the 5 time points over the course of the stress elicitation procedure. For the diurnal analysis, the cortisol values were excluded if: (1) the child took a medication known to affect cortisol on the day of saliva collection; (2) the value was >3 SDs from the mean; (3) the value was >2 SDs from the mean and either did not fit the child’s diurnal pattern and/or there was an unusual circumstance (i.e., child was reported to be “getting sick”). For the reactivity analysis, the cortisol values were excluded if: (1) the child took a medication known to affect cortisol on the day of saliva collection or (2) the value was >3 SDs from the mean.

Infant birth weight in kilograms was converted to a z-score for gestational age and sex based on national references (17). Birth weight z-scores were missing and were computed for 26 subjects using Proc Multiple Imputation in SAS. The child’s BMI measured concurrent with each saliva collection protocol was converted to a z-score based on United States Centers for Disease Control growth charts. We performed linear regression analysis to assess cortisol intercept and slope from each of the pre- and perinatal predictors described (pregnancy weight gain, pre-pregnancy BMI, infant birth weight z-score and gestational age). Linear regression analysis was also used to predict cortisol reactivity at time points 1,2,3,4 and 5 occurring before, during and after the stressor from the same set of pre- and perinatal predictors. All 7 of these models were run fully adjusted for all the covariates: child sex, child’s BMI z-score, child age in months, child’s race/ethnicity, family structure and parent’s education.

## RESULTS

As shown in Table 1, the mean age of the children was 51 months in the diurnal cortisol sample and 57 months in the cortisol reactivity sample. The sample was 50% male. Most children (60%) were white non-Hispanic. The mean pregnancy weight gain was 15.5 kg, while the mean infant gestational age was 39.4 weeks. Maternal pre-pregnancy BMI averaged 28.1 and the mean infant birth weight z-score was -0.22. Hence, the mothers were overweight before pregnancy and the children in the cohort had an average birth weight below the national references. Fourteen percent of the primary caregivers had a high school education or less. About 36% of the families were headed by a single parent.

Results of both diurnal and reactivity analyses are shown for boys and girls combined in Table 2, for girls only in Table 3 and for boys only in Table 4. We found a positive association between maternal pregnancy weight gain and diurnal cortisol intercept [β=0.20 (SE 0.007), p=0.003]. This association was more robust in girls [β=0.022 (SE 0.010), p=0.003] than in boys [β=0.017 (SE 0.009), p=0.058]. There was also a positive association between maternal pregnancy weight gain and cortisol post stress (at time point 5) in girls [β=0.002 (SE 0.001), p=0.036). Maternal pre-pregnancy BMI had no association with any of the outcomes considered. Birth weight z-score was found to have a positive association with morning cortisol [β=0.134 (SE 0.066), p=0.043]. Furthermore, we found a negative association between the child’s gestational age and cortisol time point 3 [β=-0.015 (SE 0.007), p=0.032] and this association persisted in boys [β=-0.032 (SE 0.014), p=0.027].

## DISCUSSION

To our knowledge, our study is the first to examine the independent associations of a variety of pre- and perinatal factors with cortisol diurnal pattern and reactivity to a stressor in preschool children in a single study. In addition, we believe our study is the first to test the association of pre-pregnancy BMI with these outcomes.

Our data indicated that greater maternal pregnancy weight gain was associated with higher early morning cortisol and higher cortisol during recovery from a stressor; this association was most robust among girls. This finding stands in contrast to the single prior study of pregnancy weight gain and child cortisol, which found no association of maternal pregnancy weight gain and children’s fasting plasma cortisol at age 8 years (2). This study with null findings analyzed total cortisol levels measured from a morning plasma sample, which differed from our methods. Such elevations in morning cortisol and a lack of recovery from stress may confer risk for long-term mental health and physical outcomes (8). To our knowledge, our study is the first to test the association of maternal pre-pregnancy BMI with child cortisol patterning.

In our study, the child’s birth weight z-score was positively associated with early morning free cortisol. Two prior studies found a positive association of birth weight with cortisol patterning. In one study of adults, birth weight was positively associated with total salivary cortisol (area under the curve) and bedtime cortisol (8). In the second study, among 37 newborns, lower birth weight was associated with blunted serum cortisol response to a heel stick (9). Our results differed from studies done in adults and children that found an inverse (3,4,5,6,7,8) or no association (11,12,13). There is not a clear explanation for why our study results were similar to some prior studies and not others. Differences in methodologies and sample characteristics do not suggest an explanation for the discrepant findings. However, all prior studies used absolute birth weight rather than birth weight z-score (3,4,5,6,7,8,9,10,12,13) and cortisol measurement methods were either in saliva (6,8,11), serum (2,4,5,9,12,13) or urinary cortisol metabolites (3,7). None of the studies were specifically in preschool-aged children. Future work is needed to better understand these inconsistent results.

We also found that preschool aged children, particularly boys, with higher gestational age have a lower cortisol level in response to a stressor. This finding stands in contrast to the single prior study of gestational age and stress response, which showed no association of gestational age with saliva cortisol following an adrenocorticotropic hormone stimulation test in adult males (6). These findings may have differed from ours because the age of the participants was much older than that of our cohort and the method of testing cortisol reactivity was pharmacological. Another study examined gestational age only as a moderator of the association between birth weight and free plasma cortisol, showing a positive relation between cortisol and birth weight in individuals with lower gestational age and an inverse correlation in individuals with higher gestational age (12).

To our knowledge, no prior study has examined whether the associations between pre- and perinatal factors and child cortisol patterning differ by sex. Sex differences in cortisol patterning among children have been investigated, with some studies showing no main effect of sex (18) and others showing higher cortisol among girls (19). The exact mechanism of the moderation of the associations in our study by sex is unknown.

Our findings support the observation that the prenatal environment may program the HPA axis (20). Both resting cortisol and cortisol reactivity secretion were associated with prenatal factors. Possible explanatory mechanisms include modification of the HPA axis threshold in response to stimuli from a lower to a higher set point. It has been reported that a shift seems to happen between ages 3 and 8 months from hypo- to hyperactivity of the axis in preterm infants (21). However, there are no corresponding data on term or post-term infants or older children. Hence, when exactly this shift occurs remains unknown. Furthermore, it has been recently reported that programming of the HPA axis may involve epigenetic modification of the glucocorticoid receptor (GR) gene promoter which influences tissue-specific GR expression patterns and response to stimuli (22). Maternal factors like socioeconomic status, nutrition and obesity have an effect on maternal-placental fetal endocrine and immune biology (23). Such stress can result in elevated maternal circulating glucocorticoids, which may in turn alter the related physiological processes of the offspring in the postnatal life.

Our study has several strengths. Our cohort was relatively diverse in race/ethnicity and we tested multiple pre- and perinatal factors in the same study. We controlled for the child’s age and BMI, primary caregiver’s education, child race/ethnicity and family structure. Converting birth weight to a z-score can also be cited as a point of strength in interpreting differences relating to gestational age and sex and this approach was not used in other studies. To our knowledge, our study is the first to examine pre- and perinatal factors in relation to cortisol reactivity to a behavioral stressor. There are several limitations to our study. Our study was limited to low-income preschool-aged children, which limits generalizability to other ages and other socioeconomic circumstances. Pre- and perinatal factors were gathered by maternal self-report which may include some degree of error.

Pre- and perinatal influences that confer risk for unhealthy cortisol patterning may render the child vulnerable to a variety of adverse health outcomes later in life (24). Optimal pre- and perinatal care may contribute to healthier cortisol patterning. Interventions that ameliorate the risks conferred by adverse pre- and perinatal conditions on preschool age outcomes are needed. Additional work is needed to understand the mechanisms via which pre- and perinatal factors shape cortisol diurnal patterning and cortisol reactivity to a stressor.

## Figures and Tables

**Table 1:**
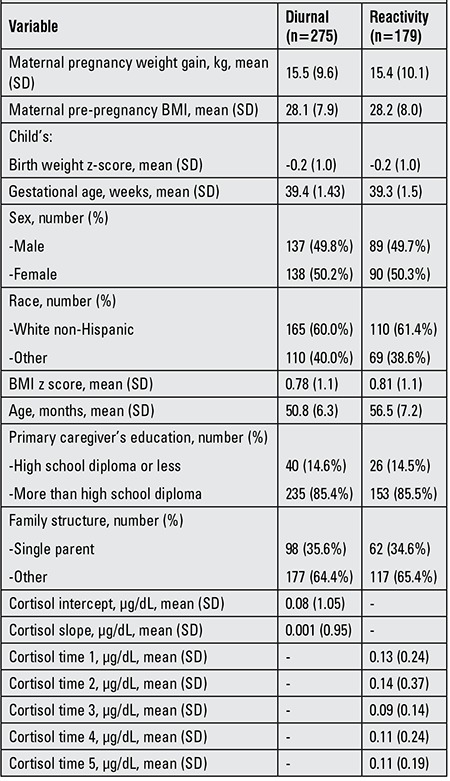
Characteristics of the sample.

**Table 2:**
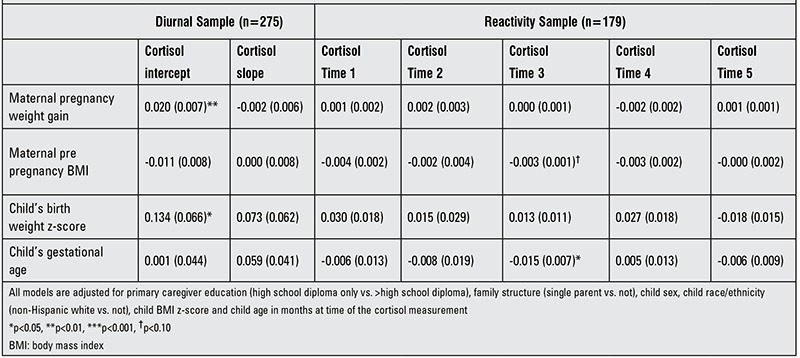
Associations between pre- and perinatal variables and cortisol diurnal patterning and reactivity to a stressor in total sample [β (SE)].

**Table 3:**
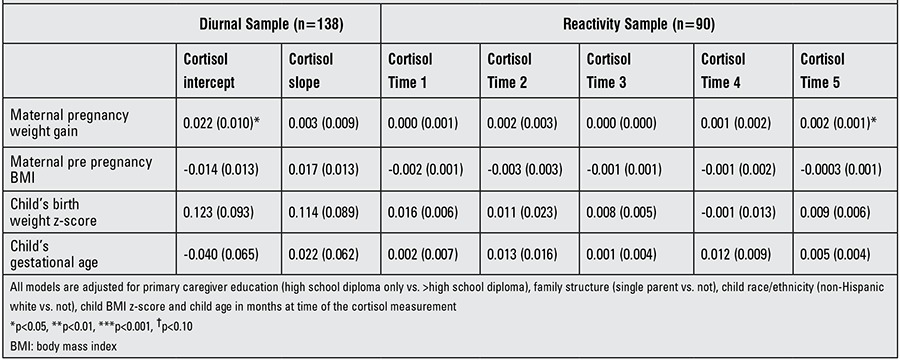
Associations between pre- and perinatal variables and cortisol diurnal patterning and reactivity to a stressor in girls [β (SE)].

**Table 4:**
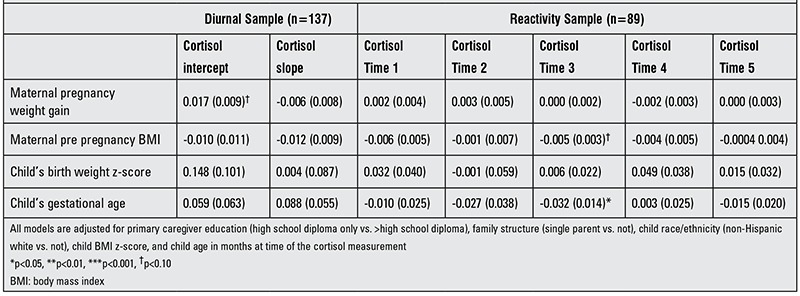
Associations between pre- and perinatal variables and cortisol diurnal patterning and reactivity to a stressor in boys [β (SE)].
